# Accidental Water Pollution Risk Analysis of Mine Tailings Ponds in Guanting Reservoir Watershed, Zhangjiakou City, China

**DOI:** 10.3390/ijerph121214983

**Published:** 2015-12-02

**Authors:** Renzhi Liu, Jing Liu, Zhijiao Zhang, Alistair Borthwick, Ke Zhang

**Affiliations:** 1 State Key Laboratory of Water Environment Simulation, School of Environment, Beijing Normal University, No. 19, Xinjiekouwai Street, Haidian District, Beijing 100875, China; 201521180038@mail.edu.cn (J.L.); zzhijiao@163.com (Z.Z.); 2 Guangdong Provincial Academy of Environmental Science, Center for Environmental Risk & Damages Assessment, Guangzhou 510045, China; 3 School of Engineering, the King’s Buildings, The University of Edinburgh, Edinburgh EH9 3JL, UK; Alistair.Borthwick@ed.ac.uk; 4 St Edmund Hall, Queen’s Lane, Oxford OX1 4AR, UK; 5 College of Civil Construction Engineering, Zhengzhou Institute of Aeronautical Industry Management, Zhengzhou 450015, China; po_drama@163.com

**Keywords:** risk analysis, mine tailings pond, heavy metal, water pollution, Guanting Reservoir, watershed

## Abstract

Over the past half century, a surprising number of major pollution incidents occurred due to tailings dam failures. Most previous studies of such incidents comprised forensic analyses of environmental impacts after a tailings dam failure, with few considering the combined pollution risk before incidents occur at a watershed-scale. We therefore propose Watershed-scale Tailings-pond Pollution Risk Analysis (WTPRA), designed for multiple mine tailings ponds, stemming from previous watershed-scale accidental pollution risk assessments. Transferred and combined risk is embedded using risk rankings of multiple routes of the “source-pathway-target” in the WTPRA. The previous approach is modified using multi-criteria analysis, dam failure models, and instantaneous water quality models, which are modified for application to multiple tailings ponds. The study area covers the basin of Gutanting Reservoir (the largest backup drinking water source for Beijing) in Zhangjiakou City, where many mine tailings ponds are located. The resultant map shows that risk is higher downstream of Gutanting Reservoir and in its two tributary basins (*i.e.*, Qingshui River and Longyang River). Conversely, risk is lower in the midstream and upstream reaches. The analysis also indicates that the most hazardous mine tailings ponds are located in Chongli and Xuanhua, and that Guanting Reservoir is the most vulnerable receptor. Sensitivity and uncertainty analyses are performed to validate the robustness of the WTPRA method.

## 1. Introduction

Industrial mining activities occur in almost every part of the world [1]. Tailing dams contain liquids (toxic, hazardous, or even radioactive) which are pollutant sources of great damage risk to humans, the environment, and ecosystems [2]. Worldwide, at least 63 major tailings dam failures were reported that caused significant pollution during 1960–2014 [3]. These tailing-dam pollution accidents accounted for 62% of the total number of major tailings dam failures during the same period. Acutely polluted water from dam breaks caused interruption of water supply (Baia Mare and Baia Borsa dam failures in Romania in 2000 [4]), human fatalities (Omai tailings dam failure in Guyana in 1995 [5]), massive fish kills (Porco tailings dam breaches in Bolivia in 1996 [6]), agricultural damage and natural reserve failure (Aznalcóllar tailings dam failure in Spain in 1998 [7]), and enormous economic loss (Tao Canyon tailings dam failure in U.S.A. in 1994 [8]). In recent years, the frequency of occurrence of such incidents has begun to shift geographically from developed countries to developing countries [9]. In P.R. China, the Ministry of Environmental Protection (MEP) responded directly to 56 reported tailing-related pollution accidents in 2006–2014 [10]. This is in the context of 11,666 mine tailings ponds that had come into operation by the end of 2013 [11]. Other major tailing-related pollution accidents in China include the Zhen’an gold tailings spill in Shaanxi Province in 2006, the Wulong gold tailings leakage in Liaoning Province in 2008, the Minjiang manganese tailings spill in Sichuan Province in 2011, and the Wantai manganese tailings leakage in Guizhou Province in 2012.

Much effort has been expended on the environmental impacts of tailings dam failures [12,13,14]. However, these previous studies focused on monitoring, managing, or remediating the fluvial environment in the immediate aftermath [5,7,15,16] or (mostly) in the long-term aftermath [4,17,18,19,20,21,22] of such failures. Few tackled pollution risk analysis and risk management before a single tailings dam failure and ensuing environmental pollution accident occurred [2,23,24], much less the accidental pollution risk analysis of multiple tailings dams (ponds) at a watershed-scale. 

We previously developed a watershed-scale accidental pollution risk assessment method [25], adopting the idea of risk ranking from the Relative Risk Model [26,27]. Following this framework, we now propose a Watershed-scale Tailings-pond Pollution Risk Analysis (WTPRA) method, which is designed for the pollution risk analysis of tailings ponds, the aim being to assist in the prevention of, or preparation for, water pollution accidents from tailings dam failures. The WTPRA method uses multi-criteria analysis, dam failure models, and instantaneous water quality models to complete a full pollution risk analysis of multiple tailings ponds at a regional-scale. The study area, Guanting Reservoir Watershed, in Zhangjiakou, accommodates over 270 metal mine tailings ponds, whose presence represents a hazard to the water catchment area of Guanting Reservoir, the largest backup drinking water resource for Beijing. We use WTPRA to analyze the pollution hazard of tailings ponds, vulnerability of receptors, and overall risk for each sub-watershed, all of which are useful for decision-making in the context of the environmental risk management of watersheds containing tailings ponds. The simulation results of tailing fluid propagation after a dam break provides key information for incident preparedness strategies and early warning systems.

## 2. Methodology and Materials

In watershed-scale accidental pollution risk assessment, existing “source-habitat-impact” risk routes are combined from multiple sources, receptors, and impacts at watershed-scale [25]. The assessment method embeds spatially cumulative impacts [28] by adding the risk ranks of multiple risk routes. Any single risk route, similar to a plausible “source-pathway-target” relationship [2,23,29], ensures a cascade of pollution hazards reaching downstream receptors. Taking advantage of the foregoing approach, we develop a Watershed-scale Tailings-pond Pollution Risk Analysis (WTPRA) method. By means of a general risk analysis process at watershed-scale [25], the WTPRA method provides detailed outputs specific to mine tailings ponds (dams).

### 2.1. Delineation of Risk Sub-Watersheds

Delineation of risk sub-watersheds involves breaking down a watershed into pollution risk regions that mark out the boundaries of the sub-areas utilized for risk analysis. This decomposition technique underpins the whole analysis process. In practice, we use the hydrological analysis function in ArcGIS tools to derive the flow networks and catchment basins from a Digital Elevation Model (DEM) of the study area [30]. Selected adjacent sub-watersheds may be amalgamated to ensure that appropriate tailings ponds, downstream rivers, and sensitive receptors (*i.e.*, humans, environment, and ecosystem) are incorporated into a given risk region.

### 2.2. Hazard Analysis of Mine Tailings Ponds

Dam failure is the worst cause of tailings fluids being released into the environment, followed by slurry pipeline failure, drainage stock or damage, piping, seepage, cracking of dam wall, and abnormal discharge. Here abnormal discharge is defined as a tailings wastewater discharge with an abnormally high volume or concentration of contaminants. The pollution hazard of a tailings pond, according to the risk source hazard approach proposed by Liu *et al.* [31], is determined by its state and control, which have been adopted into a national guideline [32]. This guideline attributes the hazard to its associated harmfulness (H) and control reliability (R), and a ranking matrix (listed in Table 1) is used to determine the hazard score. Harmfulness is classed as high, medium, and low level by considering a combination of the deleterious properties of the substances stored in the tailings pond, the storage capacity, the dam height, and the operation time. Control reliability is graded as unreliable, medium, and reliable by summing rankings related to the tailings pileup, water recycling, and susceptibility to flooding and geological hazards (e.g., earthquake, landslide, debris flow, or fracture), safety level, and preparation of emergency response [32]. Risk investigations are required to obtain data for mine tailings ponds over the entire watershed.

### 2.3. Vulnerability Analysis of Receptors

A water pollution hazard, arising from mine tailings ponds, threatens the receptors including drinking water intakes, irrigation water intakes, water bodies, residential land, and agricultural land, woodland, and nature reserves in a watershed [25]. Receptor vulnerability to tailings water pollution is ranked according to its magnitude and sensitivity using three grades, 6, 4, and 2, in accordance with national criteria or expert judgments (see Table 2).

**Table 1:** Pollution hazard ranking matrix for mine tailings pond *.

Harmfulness (H)	Reliability (R)		
	Unreliable	Medium	Reliable
High	10	8	6
Medium	8	6	4
Low	6	4	2

* Adapted from Technical Guideline for Environmental Risk Assessment of Tailings pond [32]. The scale of values is 0–10, relative and dimensionless, 10 referring to the highest and 0 to the lowest.

### 2.4. Identification of Risk Routes

**Figure 1:**
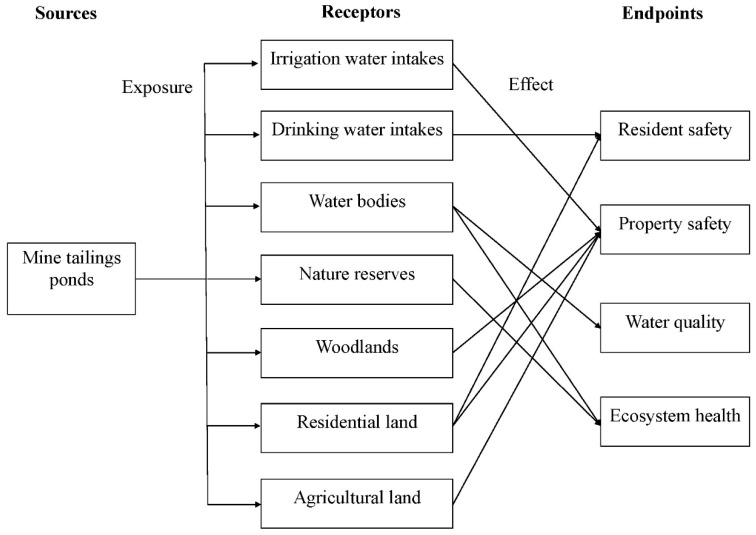
Combination of Risk Routes; adapted from Liu *et al.* [25].

A combination of multiple risk routes (see Figure 1) is identified which connects tailings ponds, receptors, pathways (exposures), and endpoints. Risk receptors mentioned in Section 2.3 are located either in a waterway (e.g., drinking water intakes) or adjacent to a waterway (e.g., agricultural land). By focusing on the water environment and its collateral values (*i.e.*, values of drinking water supply) (resident safety), irrigation water (property safety), and habitats (ecosystem health), we select resident safety, property safety, water quality, and ecosystem health as the risk assessment endpoints of tailings-related accidental water pollution. In particular, public health may be damaged by harmful tailings water coming into direct contact with human bodies in residential areas, or through humans drinking polluted water from water intakes.

**Table 2:** Vulnerability ranking criteria for risk receptors *.

Receptors	Grade	Criteria
Drinking water intakes	2	Population served ≤ 50,000
	4	50,000 < Population served ≤ 100,000
	6	Population served > 100,000
Irrigating water intakes	2	Farming area served ≤ 100 km^2^
	4	100 km^2^ < Farming area served ≤ 200 km^2^
	6	Farming area served > 200 km^2^
Residential land	2	Population density ≤ 3500/km^2^
	4	3500/km^2^ < Population density ≤ 6000/km^2^
	6	Population density > 6000/km^2^
Agricultural land	2	Green manure crops
	4	Commercial crops
	6	Food crops
Woodland	2	Shrub land
	4	Sparse woodland
	6	Forest land
Water bodies **	2	Environmental functions at grades IV and V
	4	Environmental function at grade III
	6	Environmental functions at grades I and II
Nature reserves ***	2	Experimental area
	4	Buffer area
	6	Core area

* Taken from our previous study [25]; ** According to the environmental function zones of surface water [33], grade I being for fountainhead water or national nature reserves, grade II for class 1 protective areas of centralized drinking water sources, rare aquatic habitats, fish and shrimp spawning ground, or nursery ground of larvae, grade III for class 2 protective areas of centralized drinking water sources, fishery areas such as the wintering grounds of fish and shrimp, migration channels, and aquiculture zones, or swimming zones, grade IV for industrial water or recreation water without direct contact with the human body, and grade V for the agricultural water or landscape water; *** According to the zones of nature reserves [34], a core area is an intact natural ecosystem and habitats of valuable, rare, or endangered species, with no entry permitted; a buffer area is buffering the core area, entry solely permitted for scientific researches; and an experimental area is a peripheral area of the buffer area, entry permitted for scientific experiments, practice teaching, study tours, and so on.

### 2.5. Exposure and Effect Analyses

Exposure analysis is used to screen plausible source–receptor combinations in a single risk route. Dam failures, initiating the most severe tailings fluid spills, potentially place risk receptors at the greatest exposure to tailings which may contain soluble heavy metal ions and/or toxic chemicals (such as acids and cyanide). We therefore opt to simulate the fate of tailings fluids (in case of dam failure) to help weight the probability that they propagate to the receptor. An exposure filter [27] is accordingly assigned as 0, 0.5, or 1 indicating low, medium, or high probability, respectively. Effect analysis allows us to weight the probability that exposure to the receptor causes the resulting effect to reach a given endpoint. The effect filter is assigned 0, 0.5, or 1 corresponding to low, medium, or high probability. We use a combination of a dam failure model (before the flow reaches a river) and an ensuing pollutants convection-diffusion model (after the flow discharges into the river) to simulate approximately the fate of tailings fluid and the materials contained within.

#### 2.5.1. Dam Failure Modeling

A dam failure model is constructed to simulate the routing and movement of the tailing flow discharged after the dam breach. According to Pérez-López *et al.* [13], a tailing dam may be categorized as a gravity earth dam while the tailing flow can be considered to be water containing hyper-concentrated sediment, such as observed in debris and mud flows. Here, we use a simplified form of Saint-Venant equations [35] and empirical equations commonly used in China [36,37] to simulate the dam breach. Numerical solutions are obtained for the breach width, maximum discharge at time of failure, and maximum peak value of downstream discharge.

According to historical dam failure data collected by Yellow River Institute of Hydraulic Research of China [36], in the case of a dam breach the average breach width (*b*, m) is given by

(1) $$b=K{\left(W^{\frac{1}{2}}B^{\frac{1}{2}}H\right)}^{\frac{1}{2}}$$

where *W* is the volume of water stored above breach invert at time of failure, (m^3^); *B* is the length of dam, (m); *H* is the depth of water above breach invert at time of failure, (m); $W^{\frac{1}{2}}B^{\frac{1}{2}}H\;$ represents the total energy of flow; and *K* is a coefficient related to the impact resistance quality of the dam material, generally 0.65 for clay and 1.3 for loam. Using the simplified Saint Venant equations [35], the maximum discharge of flow at time of failure (*Q_M_*, m^3^/s) takes the form

(2) $$Q_{M}=\frac{8}{27}\sqrt{g}{\left(\frac{B}{b}\right)}^{\frac{1}{4}}bH^{\frac{3}{2}}$$

where *g* is gravitational acceleration. Based on unsteady flow theory, noting the maximum discharge of flow [37], the maximum peak value of downstream routing discharge (*Q_LM_*, m^3^/s) can be empirically deduced from,

(3) $$Q_{LM}=\frac{W}{\frac{W}{Q_{M}}+\frac{L}{vk}}$$

where *L* is the distance of the tailing peak flow from the dam, (m); *v* is the maximum average velocity of flow during the flood period, (m/s); and *k* is an empirical coefficient, (s). The value of *v* can be the recorded historical maximum or simply assigned values in the ranges 1–2, 2–3, or 3–5 for plains, hilly areas, or mountainous regions, respectively. The coefficient *k* is empirically assigned 0.8~0.9, 1, or 1.1~1.5 for plains, hillsides, or mountains.

#### 2.5.2. Pollutants Convection-Diffusion Modeling

For tailing flow propagation along a river, we use the following simple instantaneous water quality model (based on convection-diffusion principles for an instantaneous point source) to simulate approximately the fate of chemicals and therefore estimate exposure probability:

(4) $$\frac{\partial C}{\partial t}+u_{x}\frac{\partial C}{\partial x}=M_{x}\frac{\partial^{2}C}{\partial x^{2}}-KC$$

The analytical solution is given by,

(5) $$C(x,t)=\int_{0}^{△t}\frac{C_{0}u_{x}}{\sqrt{4\pi M_{x}t}}\exp[-\frac{{\left(\text{x}-{\text{u}}_{\text{x}}\right)}^{2}}{4M_{x}t}]\exp(-Kt)\mathrm{dt}$$

where *t* is time, (s); *C* is the concentration of a pollutant, (mg/L); *C*_0_ is the completely mixed concentration of tailings water and river water during a prescribed time interval, (mg/L); *x* is stream-wise distance, (m); *u_x_* is the mean flow rate of the river, (m/s); *M_x_* is the longitudinal mixing or dispersion coefficient, (m^2^/s); and *K* is the rate of degradation, (d^−1^).

### 2.6. Ranking Risk of Sub-Watersheds

Following Liu *et al.* [25], a risk route score is calculated by multiplying together the hazard ranking (*H*), vulnerability ranking (*V*), exposure filter (*Ex*), and effect filter (*Ef*). The relative risk score (*RS*) of tailings-related pollution accidents in a sub-watershed is obtained by integrating all risk routes which end within the same sub-watershed. Meanwhile, the overall risk score of a tailings pond is obtained by combining all risk routes emanating from itself. Interval breaks of 150, 300, and 450 are used to rank the risk levels of the sub-watersheds as low, medium, high, and very high. The relative risk score is thus defined as

(6) $$RS_{i}=\sum H_{ij}\times V_{il}\times Ex_{jl}\times Ef_{lm}$$

where *i* is the sub-watershed series (sub-watershed 1, 2, 3, *etc.*), *j* is the tailings pond series, *l* is the receptor series, and *m* is the endpoint series.

### 2.7. Uncertainty Analysis

Uncertainty is addressed using Monte Carlo analysis, where prescribed probability distributions are assigned to risk scores and filters according to their value characteristics. The Monte Carlo simulations have been undertaken for 1000 iterations using Crystal Ball^®^ 2000 software (Decisioneering, Inc., Denver, CO, USA), and the output distributions for each sub-watershed then derived, indicating all possible risk scores and the probability of those risk scores based on the uncertainty within the model inputs or parameters. The correlation coefficients of sources, receptors, and filters in each sub-watershed have been generated by sensitivity analysis, reflecting the uncertainty results, whether influenced by model sensitivity and/or parameter uncertainty. It should be noted that the higher the correlation coefficient, the greater the contribution to the overall uncertainty.

### 2.8. Study Area and Materials

#### 2.8.1. Study Area

**Figure 2:**
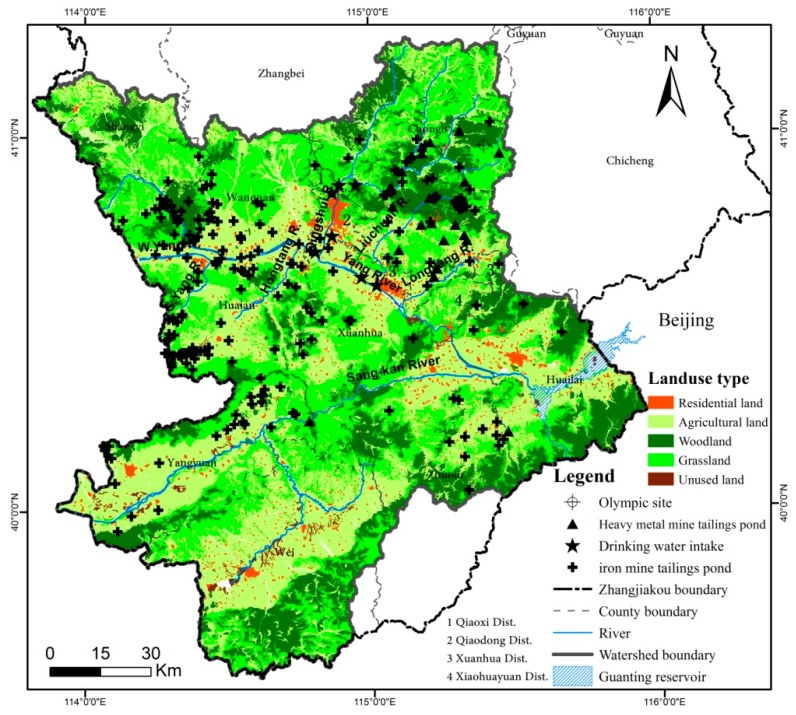
Guangting Reservoir watershed within Zhangjiakou.

Guanting Reservoir is mostly located in Zhangjiakou City, northwest Hebei Province of China (see Figure 2). The reservoir is owned by Beijing and serves as its most important backup drinking water resource. The watershed of Guanting Reservoir has a surface area of 43,402 km^2^ and is located in Inner Mongolia Province, Shanxi Province, Hebei Province, and Beijing. Three tributary watersheds, Sang-kan River, Yang River, And Guishui River, have their origins in Inner Mongolia, Shanxi Province, and Beijing, respectively. The watershed within Zhangjiakou contains the Yang River which is 192 km long and the Sang-kan River of length 175 km, and occupies a basin area of 17,965 km^2^. We selected Guanting Reservoir watershed within Zhangjiakou as the study area because it primarily affects the water catchment supplying Guanting Reservoir, and so has a major influence on the reservoir’s water security and quality. Another reason is that Zhangjiakou possesses abundant metal mineral resources (e.g., iron, gold, silver, and lead zinc), so faces a considerable acute pollution threat from many heavy metal mine tailings ponds. About 250 iron mine tailings ponds and 22 heavy metal mine tailings ponds are located within the selected area (see Figure 2), most of them along the Yang River and its biggest branch, the Qingshui River. The study area covers four administrative districts (see 1–4 in Figure 2) and nine counties (*i.e.*, Shangyi, Wangquan, Huai’an, Chongli, Xuanhua, Yangyuan, Wei, Zhuolu, and Huailai). Besides Guanting Reservoir, the fact that several drinking water intakes are situated along the mainstream channel raises the importance of risk analysis concerning local water security and water quality. Moreover, several valleys in Chongli County have been chosen to be sporting venues for the 2022 Beijing-Zhangjiakou Winter Olympic Games (24th).

#### 2.8.2. Data Sources

Three categories of data were prepared on risk sources, risk receptors, and waterways. Risk sources of mine tailings ponds (dams) were derived from two regional-scale investigation projects in Zhangjiakou City. A preliminary investigation was undertaken into tertiary prevention and control of acute water pollution risk from mine tailings ponds (dams) as part of the 12th five year plan of Zhangjiakou City during December, 2010. According to the unpublished investigation, there then existed over 520 mine tailings ponds in total in Zhangjiakou; with 488, 30, and 2 ponds for iron, heavy metal (gold, silver, or lead zinc), and coal mines, respectively. We selected the heavy metal mine tailings ponds (which potentially pose the greatest toxic water pollution threats to Guanting Reservoir and the other drinking water intakes), and proceeded with a more specialized investigation, encompassing field investigations and survey questionnaires (addressed to mining enterprises) into 22 heavy metal mine tailings ponds (within the study area, labelled T1-T22) held from July to September, 2014. Comprehensive information was obtained on each tailing pond (e.g., dam height, storage capacity, security level, operation time, and flood control criteria) with the assistance of local environmental protection bureaus. Concentrations of soluble heavy metal ions (e.g., ions of lead, mercury chrome, or cadmium) and/or toxic chemicals (e.g., acid and cyanide) were collected monthly from routine quality monitoring of tailings pond water which were conducted by the Zhangjiakou Environmental Monitoring Centre (EMC) in 2013.

Risk receptors in the Guanting Reservoir Watershed were identified as drinking water intakes (DW), residential land (RL), agricultural land (AL), woodlands (WL), and water bodies (WB). Locations and spatial distributions of land receptors (RL, AL, and WL) were derived from a 1:100,000 scale land-use map of Guanting Reservoir within Zhangjiakou (see Figure 2). This map was interpreted from the relevant remote sensing image obtained by Landsat-7 in 2010, and downloaded from the China Centre for Resources Satellite Data and Application (CRESDA) at 30 m × 30 m spatial resolution. Other information on land receptors (*i.e.*, population and crop categories) and locations, areas, and services (relating to water intakes and local water environmental functions) were obtained from Zhangjiakou City Statistical Yearbook (2013), Zhangjiakou Environmental Bulletin (2013), and data collected in 2013 by municipal bureaus of water resources, forestry, agriculture, and environmental protection. Waterway data, including the watershed boundary, were derived from a 90 × 90 m DEM, also obtained from CRESDA. The water system digital map and spatial area of Guanting Reservoir were computed by the Zhangjiakou Municipal Water Resources Bureau. Hydrological data were provided by Zhangjiakou Municipal Hydrographic Bureau. Spatial administrative boundary data were determined from the 2010 land-use map.

## 3. Results

### 3.1. The Tailings Pond Pollution Risk Map

**Figure 3:**
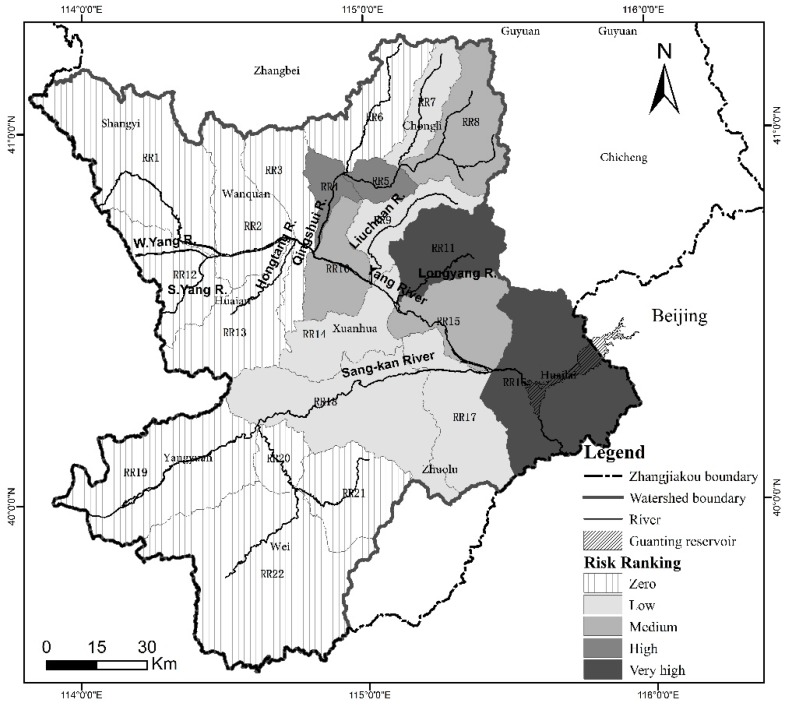
Risk ranking map for sub-watersheds RR1-22 in Guanting Reservoir Watershed within Zhangjiakou.

Taken together, the 22 sub-watersheds (labelled RR1-RR22, see Figure 3) comprise the whole Guanting Reservoir Watershed in Zhangjiakou. The Yang River and Sang-kan River basins contain 15 sub-watersheds (RR1-RR15) and 6 sub-watersheds (RR17-RR22) respectively, and both rivers eventually flow into sub-watershed RR16, a confluence area in which the Guanting Reservoir is located. All 22 heavy metal mine tailings ponds (labelled T1-T22) were identified as hazardous sources, and five categories of risk receptors (see Section 2.8.2) were analyzed in terms of vulnerability. Risk assessment endpoints comprised resident safety (RS), property safety (PS), water quality (WQ), and ecosystem health (EH) (see Section 2.4). Figure 3 shows the resulting tailings pond pollution risk map generated for Guanting Reservoir Watershed, Zhangjiakou. From the map it is evident that sub-watersheds RR11 and RR16 are at the greatest risk of acute water pollution accidents from tailings pond failures. Sub-watersheds RR4 and RR5 are at high risk; sub-watersheds RR8, RR10, and RR15 are at medium risk; and sub-watersheds RR7, RR9, RR14, RR17, and RR18 are at low risk. The remaining other 10 sub-watersheds are not at risk. These sub-watersheds, RR1-RR3, RR6, RR12-RR13, and RR19-RR22, are mostly located in the upstream parts of the selected watershed. In general, the downstream areas and two tributary basins (for the Qingshui River and Longyang River) are exposed to higher risks, the midstream reach is less at risk, and the upstream reach experiences roughly no risk.

### 3.2. Most Hazardous Tailings Ponds

**Figure 4:**
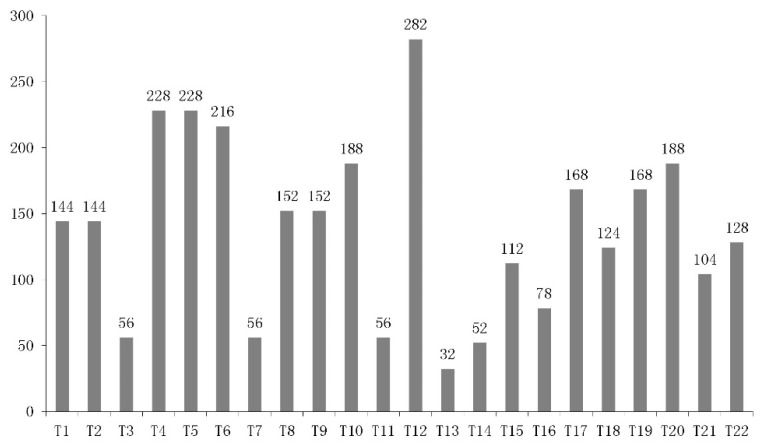
Risk score for each heavy metal mine tailings pond.

Figure 2 shows that the majority of metal mines are located in the upstream and midstream reaches of the Yang River basin. However, 250 (*i.e.*, 92%) of these are iron ore mines whose tailings water is less hazardous owing to low observed levels of heavy metal ions or other dangerous chemicals. The other 22 (*i.e.*, 8%) mine tailings ponds are the more hazardous sources, linked to the gold, silver, and lead zinc mines situated at Chongli (10), Xuanhua (10), Yangyuan (1), and Huailai (1). By overlaying the locations of the mine tailing ponds with Figure 3, it is found that ponds T1-T9 and T11-T19 are located respectively in the Longyang River and Qingshui River basins. Both rivers are branches of the Yang River. Resultant hazard rankings indicate that six heavy metal mine tailings ponds (T4-T6, T12, T16, and T18) are at medium hazard, with the remaining 16 at low hazard. Figure 4 presents the sum risk scores for each tailings pond, obtained by combining every risk route originating from a particular tailings pond. The most risky tailings ponds (risk score >200) are T4-T6, and T12; these ponds are inherently the most hazardous and/or capable of transmitting threats to more vulnerable receptors downstream. By simulating the fate of tailings fluid containing heavy metals, it was found that only a few tailings ponds (*i.e.*, T12, T17 and T19) along the Qingshui River were likely to transfer pollution threats to the mainstream of the Yang River; no threat was posed to the Guanting Reservoir by either these ponds or those along the Sang-kan River. However, the failure of eight tailings ponds/dams (*i.e.*, T1, T2, T4-T6, and T8-T9 in Longyang River and T20 in Liuchuan River) would cause serious pollution events in the Yang River and Guanting Reservoir. For Guanting Reservoir, the furthest stressor is T20 with a stream-wise distance of 118 km, and the closest is T6 with a stream-wise distance of 78 km. For example, by the time pollutants released from T4 would arrive at Guanting Reservoir, the concentrations of lead (Pb) and cadmium (Cd) would nevertheless reach 37 and 8.7 times the water quality standard (Grade III).

### 3.3. Most Vulnerable Receptors

**Figure 5:**
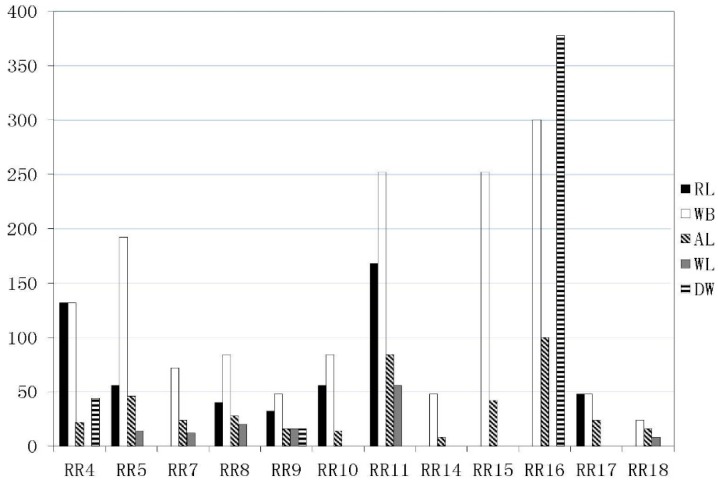
Risk score by each receptor for sub-watersheds RR1-22 (excluding no-risk sub-watersheds). The abbreviations in the legend are as follows: RL is residential land; WB is water body; AL is agricultural land; WL is woodland; DW is drinking water intakes.

Seven main underground drinking water intakes (D1-D7) serve urban areas, of which four (along the river) are plausibly susceptible to tailings pond pollution, namely: D1 in the RR4 area of the Qingshui River; D4 in RR3; D5 in RR10; and D6 in RR9 of the Yang River. The most sensitive and highest capacity drinking water intake, Guanting Reservoir, is located at the bottom of the whole catchment (RR16) (see Figure 2). Again, by simulating the fate of tailings fluid contaminated by heavy metals (see Section 2.5), it is found that: D1 is susceptible to abrupt pollution from T12, T15, and T17-T19; D4 and D5 are free from pollution threats; and D6 is exposed to T10 and T20. Guanting Reservoir is severely threatened by T1, T2, T4-T6, T8-T10, and T20 located in RR9 and R11. However, no tailings pond threatens the Olympic sites recommended in Chongli County, Zhangjiakou. Figure 5 presents the sum risk scores of sub-watersheds (excluding no-risk sub-watersheds) for each possible receptor. Water bodies (WB) are the most vulnerable receptors across the entire watershed and contribute to the majority of high risk scores. Residential land provides the next most vulnerable receptors especially in RR4, RR5, and R11 areas. In the downstream RR16 sub-catchment, the drinking water intake (DW) of Guanting Reservoir is most vulnerable to acute water pollution by heavy metal mine tailings ponds.

### 3.4. Significantly Impacted Endpoints

**Figure 6:**
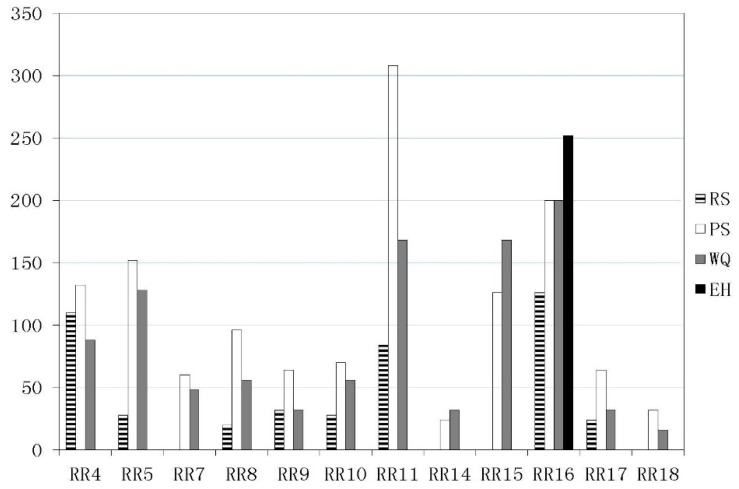
Risk score by each endpoint for sub-watersheds RR1-22 (excluding no-risk sub-watersheds). The abbreviations in the legend relate to risk assessment endpoints and are defined as follows: RS is resident safety; PS is property safety; WQ is water quality; and EH is ecosystem health.

Figure 6 shows the sum risk scores by each assessment endpoint in the sub-watersheds (excluding no risk sub-watersheds). Property safety (PS) is the most impacted endpoint over the majority of the entire watershed. Water quality (WQ) is another significantly impacted endpoint. Resident safety (RS) must be a priority, especially in the RR4, RR11, and RR16 areas. In RR16, ecosystem health is the most significantly impacted endpoint and the values of other endpoints are also quite high.

### 3.5. Uncertainty Analysis

**Figure 7:**
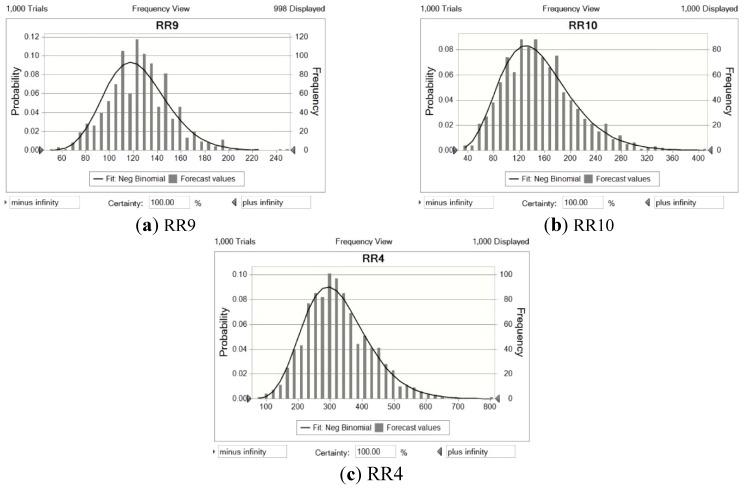
Uncertainty analysis result: risk probability distribution for region (**a**) RR9; (**b**) RR10; and (**c**) RR4.

During Watershed-scale Tailings-pond Pollution Risk Analysis, uncertainty could arise from the method itself, the choice of risk receptors, and the filters of exposure and effect. To represent sub-catchments at low, medium, and high risk, areas RR9, RR10, and RR4 are chosen for uncertainty and sensitivity analyses. Figure 7a–c presents the results of the uncertainty analysis. The distributions of the forecast are consistent with the assessment results, indicating that inherent uncertainty has not influenced the risk distribution. The wider range of the distribution implies a lower level of confidence in the risk forecast, for which RR9 (low risk region) has the highest value, and the uncertainty is higher for the upstream RR4 compared to the midstream. For RR4 and RR10, the frequency distributions are left-skewed, implying that the risk ranks may have been overestimated. Nevertheless, the distributions in RR9 have mean values that are almost the same as the risk scores, follow a normal curve, and so are estimated with a relatively accurate ranking.

**Figure 8:**
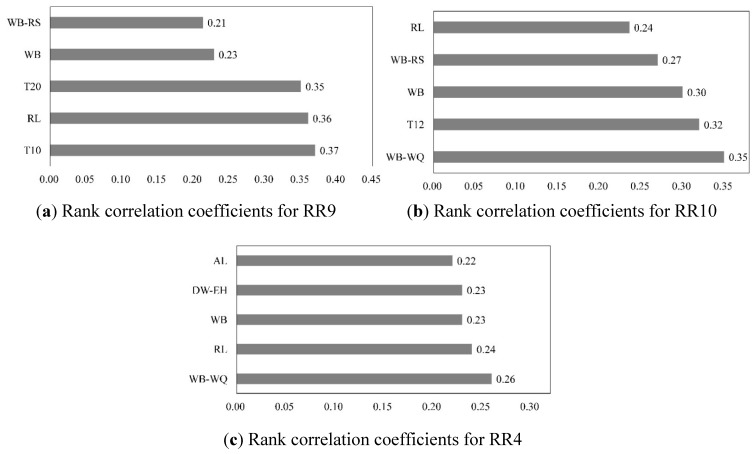
Rank correlation coefficients for different regions. The abbreviations on the y-axis relate to essential factors of assessment and are defined as follows: WB-RS and WB-WQ are effect filters of water body to residential safety and water quality, respectively; WB is water body; RL is residential land; T10 and T20 are mine tailings ponds; AL is agricultural land;. DW-EH is an effect filter of drinking water intake to ecosystem health.

Generated by sensitivity analysis, correlation coefficients for risk sources, receptors, and filters in each sub-watershed are used to represent the impact of parameter sensitivity on the uncertainty of risk assessment. The higher the correlation coefficients, the greater contribution the factors make. The highest five correlation coefficients in areas RR9, RR10, and RR4 are shown in Figure 8a–c. In the low risk sub-watershed RR9, tailings pond T10 (0.37), and residential land (0.36) contribute most to the rank correlation, followed by receptors of tailings pond T20 (0.35) and water body (0.23). As for the medium risk area RR10, the effect filters account for the highest rank correlation. For RR4, effect filters generated the uncertainty. To sum up, the uncertainty for the three sub-catchments RR9, RR10, and RR4 mainly derives from tailings ponds and effect filters.

## 4. Discussion

Watershed-scale Tailings-pond Pollution Risk Analysis (WTPRA) is designed to evaluate the transferred and combined risks arising from multiple vulnerable receptors exposed to multiple mine tailings ponds at watershed-scale. WTPRA uses risk rankings to incorporate cascading and cumulative effects into a watershed-scale risk analysis. WTPRA leads to a better understanding of the complicated interrelationships between multiple tailings ponds, receptors, and impacts. Thereby, the risk-analysis at watershed-scale provides a realistic risk map of tailings pond pollution hazards, and the most risky tailings ponds and most vulnerable receptors. Besides combining multiple tailings ponds and receptors, the new approach also incorporates upstream pollution threats into the risk analysis of any sub-catchment, which was hardly achieved in previous studies (exceptions include [2,23,25]). WTPRA assesses each tailings pond by evaluating the combined risk summed for all risk routes over a distance of 10 s or 100 s of km; this is an improvement over existing techniques such as given by China MEP [32], which recommends using a combination of tailings pond hazard and receptor vulnerability up to 10 km downstream. Moreover, WTPRA provides significant data on breach width, maximum peak discharge, and temporal-spatial concentrations of pollutants in the stream-wise direction, using a combination of dam failure and pollutant convection-diffusion models. These quantitative data are helpful not only to screen a risk route and assign exposure filters, but also to develop a mitigation/emergency plan of point-to-point countermeasures in terms of locations, arrival times, and pollutant concentrations.

Following the standardized process given by Liu *et al.* [25], WTPRA undertakes a six-step risk ranking procedure (see Section 2), in accordance with strict standards and criteria. Using WTPRA, the watershed risk related to acute water pollution of tailings ponds could be estimated and compared for different watersheds in a single exercise, following common ranking criteria. The Guanting Reservoir basin case study demonstrates that WTPRA is both operable and applicable. 

From the resultant risk map, sub-catchment areas RR11 and RR16 are at very high risk. RR11 is exposed to risk due to the proliferation of heavy metal mine tailings ponds, which could potentially release heavy metals and threaten the safety of local water bodies, residential, and agricultural land (see Figure 2 and Figure 5). RR16 contains the largest receptors of Gutaning Reservoir, which is most susceptible to transferred pollution events upstream of the RR11 and RR9 areas, which could seriously endanger drinking water supply and ecosystem health (see Figure 5 and Figure 6). The tributary sub-catchment areas RR5 and RR4 are at high risk, because many upstream and local tailings ponds pose acute pollution hazards to water bodies in the RR5 area (see Figure 2), and subsequently threaten a densely populated urban area (Qiaoxi District) and a main drinking water intake (D1) in RR4 (see Figure 5). Areas RR15 and RR10 are at medium risk of transferred pollution from the upstream reaches, RR8 is also at medium risk because of the presence of local tailings ponds. The low-risk sub-catchment areas are barely affected by tailings pond pollution events. The rest of the sub-catchment area is a no-risk region, mostly located in sub-catchments at the upstream reaches of Yang River and Sang-kan River where no heavy metal mine tailings ponds are situated.

The risk analysis results indicate that T4-T6 and T12 are the most hazardous tailings ponds due to high harmfulness, even though the dams are believed to be reliably controlled. Each pond has a storage capacity exceeding 0.5 million m^3^ with a dam at least 30 m high. T16 and T18 are also hazardous because of moderate harmfulness and medium control reliability, both of which correspond to an unsafe tailings pond. The other 16 tailings ponds are at low hazard because they are well managed and remain less harmful. T4-T6 and T12 are the most risky tailings ponds in the entire watershed because they are most hazardous and would have most impact should they fail (risk score >200). For the D1 drinking water intake, the simulation predicts that T12, T15, and T17-T19 are more hazardous, with the highest concentration of Cd exceeding four times the water quality standard (grade III) and a minimum arrival time of 26 h over the shortest distance of 19 km. For the D6 drinking water intake, T10 and T20 could cause events where the concentrations of Pb and Cd reach 4 and 10 times the standard in less time. For Guanting Reservoir, the more hazardous tailings ponds are T1, T2, T4-T6, T8-T10, and T20; in particular, T4 leads to the highest concentrations of pollutants (see Section 3.2). It would take somewhere between 89 and 107 h for contamination to arrive at the reservoir, once tailings pond water is released into the Yang River.

Based on the aforementioned analysis, countermeasures are recommended concerning the most affected areas, the most hazardous tailings ponds, and the most vulnerable receptors. For the entire watershed, priority is given to the high risk sub-catchment areas (RR4, RR5, RR11, and RR16) in terms of risk prevention and mitigation. Monitoring sections for early-warning systems are therefore suggested for the outlets of Qingshui River, Liuchuan River, and Longyang River, and upstream sections close to drinking water intakes (D1, D6, and Guanting Reservoir). Another measure would be to construct multiple cascades of intercept dams along larger river branches, such as the Liuchuan River, Longyang River, and mainstream, east branch, and middle branch of the Qingshui River. Preparation of an intercept dam involves site selection, storage of building materials (sand, stone, and concrete), and storage of intercepting materials (sandbags, cement pipes, and filter boxes of sodium sulphide, chlorinated lime, or active carbon). For major tailings ponds, the most important measure is to maintain the pond/dam so that it remains safe. T16 and T18 urgently require reinforcement. Proper risk management procedures and additional engineering buildings, cofferdams and accident pools, are recommended for all major tailings ponds. Emergency response measures such as emergency monitoring, pumping tailings fluid, and neutralizing pollutants, need to be planned and workable especially in T4-T6 and T12. It is also strongly suggested that detention ponds should be constructed (if the local topography is suitable) at inlets to the main river branches for ponds located close to Guanting Reservoir (*i.e.*, T1, T2, T4-T6, T8-T10, and T20). For major receptors, noting the longer arrival time of pollutants, a sufficient response plan should be devised. Construction of a large detention pond is recommended at the upstream reach of sensitive receptors, particularly for D1, D6, and Guanting Reservoir. Corresponding storage of neutralizing chemicals and preparedness for emergency monitoring are needed in the case of detention pond use. 

It should be noted however, that WTPRA may have overestimated the risk in certain areas of the Guanting Reservoir Watershed, as indicated by the results of the uncertainty analysis. One reason for this is that the relatively low quality of data collected has not been properly represented by either the simulation process or risk analysis. It is quite possible that routine monitoring provided incorrect estimates of the concentrations of soluble heavy metal ions (e.g., Pb of 0.2 mg/L and Cd of 0.05 mg/L) and cyanide in tailings water. Scarcity of daily hydrological data led to an annual format being used instead. It is recommended that a more comprehensive data collection campaign be conducted in the future.

## 5. Conclusions

A WTPRA approach was proposed for risk analysis of mine tailings pond pollution at watershed-scale. The approach modified a previous watershed-scale accidental pollution risk assessment method and then customized it for mine tailings ponds. A set of common criteria was constructed to rank the hazards from different mine tailings ponds and the vulnerability of receptors. A combination of a dam failure model and an instantaneous water quality model was utilized to simulate the fate of chemically contaminated tailings fluid, and quantify the exposure probability. By embedding cascading effects and spatially cumulative effects, a comprehensive risk analysis was achieved for mine tailings pond pollution at a watershed-scale. The resultant risk map for the case study of the Guanting Reservoir basin indicated that the downstream sub-catchment and two tributary basins (*i.e.*, Qingshui River and Longyang River) are at a higher risk than the midstream and upstream sub-catchments. The most hazardous mine tailings ponds are mainly concentrated in the mountainous areas of Chongli and Xuanhua. Tailings ponds T4-T6 and T12 pose greatest risk due to the higher hazard and more vulnerable receptors affected. The most vulnerable receptors involve the water environment, in particular Guanting Reservoir and the drinking water intakes, D1 and D6. Guanting Reservoir is exposed to the T1, T2, T4-T6, T8-T10, and T20 tailings ponds. Engineering (e.g., cofferdam, accident pool, interception dam, and detention pond) and management (monitoring, reinforcement, and emergency planning) countermeasures are required to address the most affected areas, the most hazardous tailings ponds, and the most vulnerable receptors. The analysis results provide useful information for risk planning and daily management of mine tailings ponds in terms of risk prevention and mitigation, incident-preparedness strategies, and early warning systems. Risk uncertainty in the WTPRA would be reduced by improving the quality of data collected.
